# Expression of a urokinase‐type plasminogen activator during tumor growth leads to angiogenesis via galanin activation in tumor‐bearing mice

**DOI:** 10.1002/2211-5463.12318

**Published:** 2017-10-09

**Authors:** Hiroyuki Yamamoto, Rina Okada, Rika Tanaka, Keiko Unno, Kazuaki Iguchi

**Affiliations:** ^1^ Nihon Pharmaceutical University Ina‐machi, Kitaadachi‐gun Japan; ^2^ Laboratory of Bioorganic Chemistry School of Pharmaceutical Sciences University of Shizuoka Japan

**Keywords:** adhesion molecule, extracellular processing, galanin, neuropeptide, plasminogen activator, tumor growth

## Abstract

Small‐cell lung carcinoma releases progalanin. The released progalanin is activated via a nonclassical processing pathway, being processed into an active form of galanin (1–20) by plasmin in extracellular components. Plasmin is produced from plasminogen activators. To clarify the regulation of progalanin via plasminogen activation by urokinase and tissue‐plasminogen activator (t‐PA), we investigated the regulation mechanism for urokinase and t‐PA expression and their effect on galanin activation. Additionally, we studied the effect of activated galanin on angiogenesis. To determine the effect of cell density, we measured the expression levels of urokinase and t‐PA using real‐time PCR and plasminogen/gelatin zymography in a cell culture. The urokinase expression increased under both high cell density and presence of cell membrane fractions. However, urokinase increments induced by conditioned medium were low. These results indicate that expression of plasminogen activators is regulated by cell membrane factors. We used tumor‐bearing mice to clarify the expression of plasminogen activators and galanin activation. Real‐time PCR showed that urokinase was substantially higher in the central parts of tumors compared to the periphery, and this was confirmed by plasminogen/gelatin zymography. To evaluate the biological effect of plasminogen activators on tumor growth, we used tranexamic acid as a plasminogen inhibitor. Tranexamic acid decreased galanin (1–20) and the hemoglobin content of tumors and suppressed tumor growth. Additionally, galanin had no effect on the hemoglobin content of tumors derived from cells lacking GALR2. These results demonstrate the regulation of urokinase expression in tumors through progalanin activation in extracellular compartments, and confirm that galanin plays a role in angiogenesis.

**Abbreviations**
Hb: hemoglobin
HRP: horseradish peroxidase
MMP: matrix metalloproteinase
RIA: radioimmunoassay
SCLC: small‐cell lung carcinoma
t‐PA: tissue‐plasminogen activator
u‐PA: urokinase

Tumors are regulated by several mechanisms, such as growth factors, tumor factors, and hormonal peptides. The tumor tissue environment continues to change during tumor growth 1. Angiogenesis in particular requires control because tumor growth requires many essential nutrients and oxygen. Tumors therefore induce angiogenesis via VEGF 2, 3, bFGF 4, angiopoietin 3, matrix metalloproteinase (MMP) 5, and plasmin 5, making angiogenesis an important target in suppressing tumor growth and metastasis.

Plasmin, a serine protease, is a tryptic protease. There are many reports on the effects of plasmin on tumor growth, invasion, and metastasis, particularly its effect on angiogenesis and cell migration 5, 6. Plasmin is converted from plasminogen by two plasminogen activators, urokinase (u‐PA) and tissue‐plasminogen activator (t‐PA) 7. Some cancer cells produce u‐PA and there is a positive correlation between the amount of u‐PA expression and malignancy 8, 9.

Some ectopic hormone‐producing tumors express neuropeptides 10, 11, 12, 13, 14, 15. However, lack of prohormone convertase and/or constitutive release was found to cause the release of these neuropeptides in a precursor form 16, 17, 18. In addition, there are many cases where these ectopic hormone‐producing tumors express their hormone‐specific receptors 19, 20, 21, 22, 23, 24. However, precursor peptides usually show a low binding potential to their own specific receptors. Our previous studies show that small‐cell lung carcinoma (SCLC) produced and released progalanin, a galanin precursor, and that plasmin converted progalanin to an active form of galanin (1–20) 25, 26, 27. In addition, the activation mechanism of progalanin induced angiogenesis via incrementation of MMP expression 27. This activation mechanism is different from a classical peptide processing pathway, so there exists little evidence regarding the activation of precursor peptides in the extracellular environment. In this study, we aimed to clarify expression of plasminogen activators during tumor growth and the effect of plasminogen activators on progalanin activation. In addition, we studied the effect of activated galanin on angiogenesis.

## Materials and methods

### Cell culture

Human SCLC cell lines SBC‐3A 27 and SBC‐3A‐Y were used. The SBC‐3A cells expressed the type 2 galanin receptor (GALR2), while the SBC‐3A‐Y cells were isolated from a subclone that lacked GALR2 (Figs S1 and S2). These cells were cultured in RPMI‐1640 medium (Nissui Pharmaceutical, Tokyo, Japan) supplemented with 10% (v/v) FBS (Moretate Biotech, Bulimba, Australia) in a humidified atmosphere of 5% CO_2_ and 95% air at 37 °C. When cells reached 80% confluency, they were dispersed with 0.05% (w/v) trypsin in phosphate‐buffered saline (PBS) and harvested at a concentration of 10^4^ cells·mL^−1^.

### Animals

Animal experimental protocols were approved by the ethics committee of the University of Shizuoka and performed in accordance with the guidelines for the Care and Use of Laboratory Animals of the University of Shizuoka. Male KSN/slc mice were purchased from Nippon SLC Ltd. (Shizuoka, Japan) and housed under standard laboratory conditions (23 ± 1 °C, 55 ± 5% humidity) with access to tap water and food *ad libitum*. Lights were automatically turned on at 08:00 h and off at 20:00 h.

### Expression of plasminogen activators depends on cell density

The SBC‐3A cells were seeded in culture dishes at densities of 10^4^, 10^5^, or 10^6^ cells/10 cm^2^. After 24 h, the culture media and cells were collected. The culture media were used to assess plasminogen activator activity with fluorescent substrates and plasminogen/gelatin zymography. The cells were used to measure plasminogen activator mRNA expression, via real‐time PCR.

### Regulation of expression of plasminogen activators by conditioned media and cell membranes

Conditioned media were prepared as follows: 50% confluent SBC‐3A cells were cultured for 48 h, after which the cultured media were centrifuged at 3000 ***g*** for 30 min. Supernatants were stored at −20 °C and used as conditioned media. The cell membranes were prepared as described below. Briefly, SBC‐3A cells were collected with a scraper. The cells were homogenized using a Teflon glass homogenizer. The homogenate was centrifuged at 1000 ***g*** for 30 min and the supernatant collected. The supernatant was centrifuged at 30 000 ***g*** at 4 °C for 30 min, and the resulting pellets were used as the cell membrane fraction.

SBC‐3A cells were cultured in media containing the conditioned media or the cell membrane fractions for 24 h, after which the cultured cells were collected. The plasminogen activator mRNA expression in the cells was measured via real‐time PCR.

### Expression of plasminogen activators in tumors

Tumor samples were obtained as previously described 28. Briefly, KSN/slc mice were implanted with SBC‐3A cells (1 × 10^6^ cells/100 μL Matrigel) subcutaneously on the dorsal side. When the tumors reached a diameter of 7–10 mm, they were excised. The tumors were separated into two parts: central and peripheral. For hemoglobin content measurement, western blot analysis, and plasminogen/gelatin zymography, the samples were homogenized in a protein extraction buffer (500 mm Tris/HCl, pH 6.8, 0.1% Triton X‐100) using a Polytron homogenizer. The homogenates were centrifuged at 3000 ***g*** for 30 min, and the supernatant was used as the tumor extract. The protein concentration of the extract was measured using the Coomassie Brilliant Blue method, with BSA as the standard. For real‐time PCR analysis, total RNA was extracted from the tumor with TriPure Isolation Reagent (Roche, Basel, Switzerland).

### Preparation of tumor extracts for gel filtration chromatography

KSN/slc mice were implanted with SBC‐3A cells (1 × 10^6^ cells/100 μL Matrigel) subcutaneously on the dorsal side. To suppress plasmin activity, tranexamic acid was administered intraperitoneally at a dose of 30 mg·day^−1^. When the tumors reached a diameter of 7–10 mm, they were excised. To determine the molecular forms of galanin, the collected samples were heated in a boiling water bath for 10 min in 0.1 m acetic acid. After cooling, the acetic acid concentration was increased to 1 m and samples were homogenized in a Teflon pestle homogenizer. The homogenates were then centrifuged at 3000 ***g*** for 30 min. Supernatants were lyophilized and used as tumor extracts. Tumor extracts were eluted on a Sephadex G‐50 fine column (1.0 × 100 cm, GE Healthcare UK, Chalfont St Giles, UK) using 1 m acetic acid as the eluent. The eluate was collected in 0.9 mL volumes and lyophilized. The lyophilized fractions were dissolved in the standard RIA diluent. The column was calibrated with BSA (molecular weight 69 kDa, void volume), lysozyme (14.4 kDa), human galanin (3 kDa), and dibutyryl cAMP (total volume).

### The effect of type 2 galanin receptors on tumor growth

KSN/slc mice were implanted with SBC‐3A or SBC‐3A‐Y cells (1 × 10^6^ cells/100 μL Matrigel) subcutaneously on the dorsal side. To suppress plasmin activity, tranexamic acid was administered intraperitoneally at a dose of 30 mg·day^−1^. Stimulation galanin was injected, at a volume of 50 μL and a concentration of 50 ng/50 μL in saline, directly into the tumor. We used a saline control. Ten days after the cells had been implanted, the tumors were collected, weighed, and extracted using homogenizer, for the measurement of the hemoglobin content.

### Real‐time PCR

Total RNA was extracted from SBC‐3A cells and tumors with TriPure Isolation Reagent (Roche, Basel, Switzerland). Genomic DNA was removed with DNase I. cDNA was synthesized using ReverTra Ace (Toyobo, Osaka, Japan) according to the manufacturer's instructions. Specific primers were designed to amplify human t‐PA (sense: GAGGACCAGGGCATCAGCTA; antisense: CGTGCCCCTGTAGCTGAT), human u‐PA (sense: CACCACCATCGAGAACCAGC; antisense: CGGTGCCTCCTGTAGATGG), and human β‐actin (sense: GCGGGAAATCGTGCGTGACATT; antisense: GATGGAGTTGAAGGTAGTTTCGTG). Real‐time PCR was performed with Thunderbird SYBR qPCR Mix (Toyobo) using LightCyclerNano (Toyobo) and StepOne systems (Thermo Fisher Scientific, Waltham, MA, USA). The PCR products were evaluated using melting curve analysis.

### Radioimmunoassay

Radioimmunoassay (RIA) was performed at 4 °C as described previously 29. R0672 antibodies specific to the N‐terminal region of galanin were raised in rabbits against synthetic human galanin (1–15).

### Western blot analysis

Samples (5 μg) of the cell lysates were separated on a 15% (w/v) polyacrylamide gel 30. Proteins were blotted onto a nitrocellulose membrane (Protran BA85, GE Healthcare UK) in a semidry blotting system (NA‐1513, Nihon Eidoh, Japan) 31. Nitrocellulose membranes were blocked with 1% (w/v) BSA. Blocked membranes were incubated with anti‐HIF‐1α antibody for HIF‐1α (× 1000, Bethyl Laboratories, Montgomery, TX, USA), then with horseradish peroxidase (HRP)‐conjugated anti‐rabbit IgG goat antibody (Biosource, Camarillo, CA, USA). The blots were subsequently developed on a chemiluminescent detection system 32 or Imobiron (Merck Millipore, Billerica, MA, USA) using LuminoGraph (Atto, Amherst, NY, USA). The bands were densitometrically analyzed using cs analyzer software (Atto corp., Tokyo, Japan).

### Plasminogen/gelatin zymography

We measured plasminogen activator activity using plasminogen/gelatin zymography 33. The tumor extracts were separated with 10% polyacrylamide gel containing plasminogen and 1 mg·mL^−1^ gelatin. After electrophoresis, the SDS in the gels was removed with Tris/HCl buffer (pH 7.4) containing 2.5% Triton X‐100, then incubated in Tris/HCl containing 12.5 mm MgCl_2_ buffer for 24 h to allow digestion of the gelatin. The gels were visualized using Coomassie Brilliant Blue R250.

### Detection of plasminogen activator‐like enzyme activity by Pyr‐Gly‐Arg‐MCA cleavage assay

We determined plasminogen activator‐like enzyme activity in cell extracts using a Pyr‐Gly‐Arg‐MCA (Peptide Institute, Osaka, Japan) cleavage assay as follows. The cell extracts were diluted in 50 mm Tris/HCl buffer (pH 7.4), after which Pyr‐Gly‐Arg‐MCA (final concentration 100 μm) was added to the solution, including the extracts. The solution was incubated at 37 °C for 2 h, and cleaved 7‐amino‐4‐methyl coumarin was measured (excitation/emission, 355/460 nm).

### Hemoglobin content in tumor

We assessed angiogenesis by measuring hemoglobin (Hb) content. Hb was measured by a slightly modified cyanmethemoglobin method 34. The tumor extracts from the Matrigel plugs were diluted with 100 μL of 0.5 mm sodium hydroxide, to which were added 20 μL of 2% (w/v) potassium ferricyanide and 20 μL of 0.5% (w/v) sodium cyanide. After 30 min of incubation, Hb concentrations were determined by measuring absorbance at 550 nm.

### Data analysis and statistics

Data are represented as means ± SEM. Tukey's tests were used for statistical analysis program (R).

## Results

### Expression of plasminogen activators in culture cells

**Figure 1:**
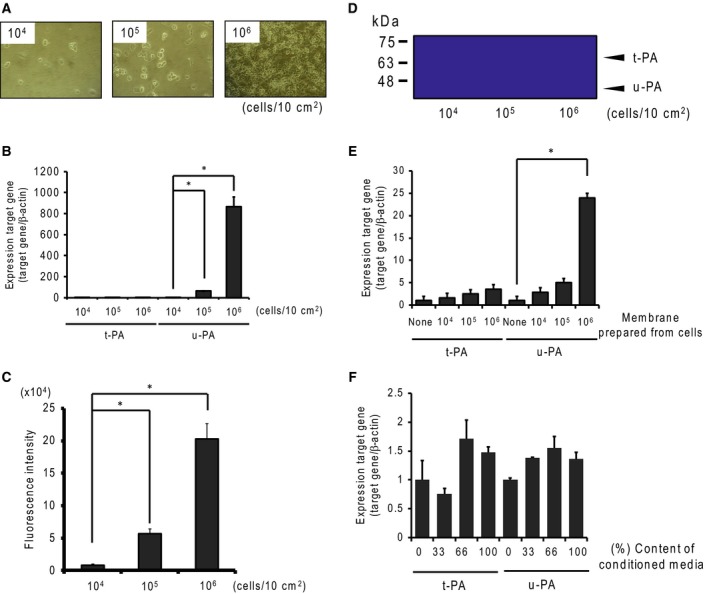
Induction of plasminogen activators in SBC‐3A cells. (A) SBC‐3A cells were seeded in a culture dish at a density of 10^4^, 10^5^, or 10^6^ cells/10 cm^2^. (B) The effects of cell seed density on plasminogen activator mRNA expression were determined by real‐time PCR analysis. Cells seeded at several densities were cultured for 24 h, and RNA was isolated. Expression of u‐PA and t‐PA mRNA was measured by real‐time PCR analysis. β‐Actin mRNA was used as a reference gene. *N* = 3–4, *P < 0.05 vs. 10^4^ cells. (C) Plasminogen activator activity was measured using fluorescent substrate (Pyr‐Gly‐Arg‐MCA). The substrate was digested with culture medium, after which the digest was measured by fluorescence (355 nm/460 nm). *N* = 3–4, *P < 0.05 vs. 10^4^ cells. (D) Plasminogen activator activity and molecular forms were determined by plasminogen/gelatin zymography. Samples of 50 μg per lane were loaded for electrophoresis, using 10% acrylamide gel containing 1% gelatin and 50 μg·mL^−1^ plasminogen. t‐PA and u‐PA were detected at ~70 kDa and ~50 kDa, respectively. The effect of cell membrane fraction (E) and conditioned media (F) on plasminogen activators mRNA expression was determined by real‐time PCR analysis. *N* = 3–4, *P < 0.05.

We determined the effect of cell seed density on u‐PA and t‐PA expression using real‐time PCR analysis, protease activity, and plasminogen/gelatin zymography. The mRNA contents of u‐PA increased gradually with cell density (Fig. 1B). Additionally, the expressions of u‐PA and t‐PA proteases were measured by fluorescent substrate and plasminogen/gelatin zymography analysis. We detected protease activity of fluorescent substrate from the high sensitivity and total enzymatic activity of u‐PA and t‐PA. Plasminogen activity gradually increased with cell density (Fig. 1C). In plasminogen/gelatin zymography, plasminogen in the gels was found to be activated where u‐PA and t‐PA were present. We detected u‐PA and t‐PA at about 50 kDa and 70 kDa, respectively (Fig. 1D). Accumulated plasminogen activation was found to be similarly activated, especially in the presence of high amounts of u‐PA. We then explored what factors regulated u‐PA and t‐PA expression, focusing on cell adhesion and/or tumor factors. We assessed the effects of cell adhesion via coculturing with cell membranes prepared from SBC‐3A cells and evaluated tumor factors using SBC‐3A cells in culture‐conditioned media. Membranes were prepared from cultured cells at a density of 10^4^, 10^5^, or 10^6^ cells/10 cm^2^. The expression of u‐PA induced by cell membranes, particularly membranes prepared from 10^6^ cells, increased significantly (Fig. 1E). Conditioned media also tended to induce u‐PA expression, although to a lesser degree (Fig. 1F). These results indicate that the u‐PA‐inducing factor exists in cell membranes and the extracellular environment, such as between‐cell adhesion, controls expression of u‐PA‐inducing factors.

### Hemoglobin content and expression of HIF‐1 and u‐PA in tumor tissue

**Figure 2:**
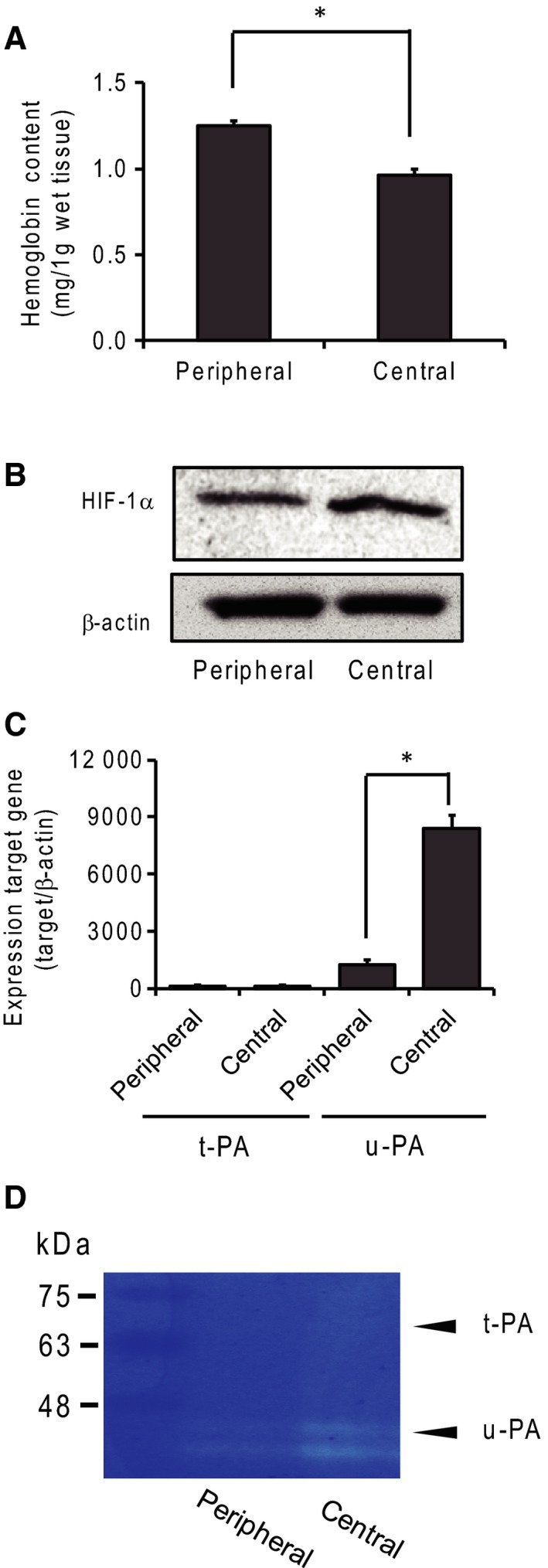
Expression of plasminogen activators in tumor tissue. (A) Hemoglobin content in the central and peripheral tumor regions. Hemoglobin content was measured with the cyanmethemoglobin method and found to be related to angiogenesis in tumor regions. *N* = 4, *P < 0.05. (B) HIF‐1α expression was detected by western blotting analysis under hypoxic conditions. (C) Expression of t‐PA and u‐PA mRNA in the peripheral and central tumor regions was measured by real‐time PCR. *N* = 4, *: *P* < 0.05. (D) The expression of plasminogen activators was determined by plasminogen/gelatin zymography.

To evaluate the hypoxic environment, we measured hemoglobin content and HIF‐1 expression. Hemoglobin content was correlated with the number of blood vessels, and HIF‐1 expression was induced under hypoxic conditions. Tumors reaching 7–10 mm were separated into central and peripheral regions, and we collected approximately 2 mm^2^ of tumor tissue from each region. The hemoglobin content of the central region was lower than that of the peripheral region, indicating a lower number of blood vessels in the central region (Fig. 2A). In addition, expression of HIF‐1α in the central region was slightly higher than that in the peripheral region (Fig. 2B). The densitometry analysis of the ratio of HIF‐1α to actin showed that the density of the central region was 1.4 times that of the peripheral region. These results indicate that the central and peripheral parts of the tumor differ in extracellular environmental conditions, such as oxygen saturation and nutrition.

To determine the expression of plasminogen activators in tumors, we performed real‐time PCR and plasminogen/gelatin zymography. A higher expression of u‐PA mRNA than of t‐PA mRNA was detected in both tumor regions (Fig. 2C). In addition, the expression of u‐PA mRNA was about sevenfold higher in the central than in the peripheral region. Enzymatic activity was detected by plasminogen/gelatin zymography. The major plasminogen activator was u‐PA, and t‐PA expression was low. In addition, u‐PA expression was higher in the central than in the peripheral region (Fig. 2D).

### The effect of plasminogen activators on galanin activation and tumor growth

**Figure 3:**
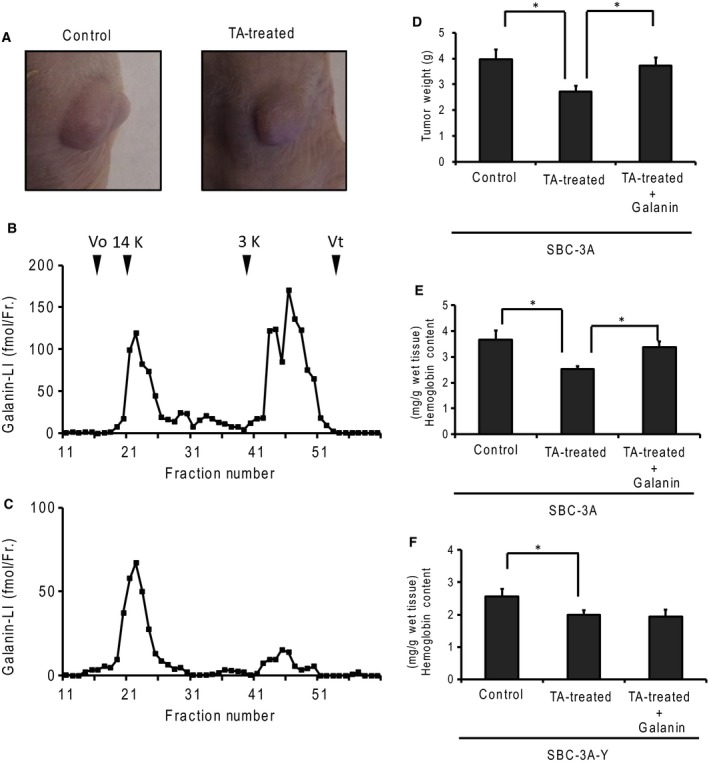
Involvement of plasminogen activators in galanin production and tumor growth. (A) Tumors generated in tumor‐bearing mice. SBC‐3A cells were injected into the subcutaneous layer. The tumor was grown to a diameter of 7–10 mm. To suppress plasmin activity, the mice were administered tranexamic acid (TA). The molecular forms of galanin‐like immunoreactivity in the tumor (B) were treated with tranexamic acid (C) using gel filtration. The gel was carried by Sephadex G‐50 fine (1.0 cm × 80 cm) and 1 m acetic acid as an eluent. The column was calibrated with bovine serum albumin (Vo), lysozyme (14 kDa), human galanin (3 kDa), and dibutyryl cAMP (Vt). (D) Tumor growth was measured by tumor weight at 10 days after implanting SBC‐3A cells into the subcutaneous layer. The mice were treated with TA (30 mg·day^−1^, intraperitoneal administration) to suppress plasmin activity. *N* = 4, *P < 0.05. (E and F) Hemoglobin content was measured in tumors derived from SBC‐3A or SBC‐3A‐Y cells, using the cyanmethemoglobin method. Hemoglobin decreased with TA treatment, but recovered under galanin administration (1 μg·day^−1^, direct injection into tumor). *N* = 4, *P < 0.05.

We evaluated tumor growth by measuring cell diameter after implanting small‐cell lung carcinoma cells mixed in Matrigel (Fig. 3A). We used tranexamic acid, a plasmin inhibitor, to evaluate galanin activation and tumor growth. Galanin activation was evaluated using a molecular form of galanin‐like immunoreactivity. The molecular forms of galanin‐LI were determined using gel filtration chromatography. The galanin‐LI in tumor‐bearing nude mice existed in a ~2‐kDa form (Fig. 3B). In the tranexamic acid‐treated mice, the amount of 2‐kDa molecular forms of galanin‐LI decreased and the 12‐kDa forms of galanin‐LI, indicating the presence of the progalanin form, increased relative to it (Fig. 3C). Tumors continued to grow over time. Tranexamic acid treatment inhibited tumor growth by about 30% relative to the nontreated group (Fig. 3D). Hemoglobin content, which correlates with the number of blood vessels, also decreased following tranexamic acid treatment (Fig. 3E). Tumor growth and hemoglobin content recovered following the application of galanin directly to the tumor. In contrast, hemoglobin content did not recover in the tumors originating from SBC‐3A‐Y cells, which were the SBC‐3A subclones lacking the type 2 galanin receptor (Fig. 3F).

## Discussion

The plasminogen activation system is composed of two plasminogen activators, t‐PA and u‐PA 7. This system plays an important role in tumor growth by affecting extracellular matrix digestion, matrix metalloprotease activation, and growth factor activation 5, 6. The plasminogen system is essential for angiogenesis in hypoxic environments. It is well known that oxygen and nutrient supply was important during tumor growth because tumor cell activity was high and tumor cells were actively dividing. Although tumor expansion led to hypoxia and malnutrition owing to increasing diffusion distances from vascular tissue and tumor cells consuming oxygen and nutrients, tumors required new vessel formation, thus supporting cell division. There are many reports connecting tumor growth and angiogenesis. The plasminogen system is a well‐known angiogenesis mechanism involved in tumor growth 35, 36. u‐PA in particular was found to be widely expressed in several cancer cells and to play a role in tumor progression, invasion, and metastasis 8. In the present study, we demonstrated the regulation of plasminogen activator expression using *in vitro* and *in vivo* approaches. In the SCLC cell line SBC‐3A, which also expresses u‐PA mRNA, the expression of u‐PA mRNA and u‐PA‐like enzyme activity increased with cell density and tumor factors. We verified this via an *in vivo* study using SCLC tumor‐bearing model mice. We prepared the central part of the tumor tissue as a hypoxic and malnourished region and the peripheral part under normal conditions. This resulted in higher u‐PA expression in the central than in peripheral tumor region. The mechanism behind this induced u‐PA expression is unclear, but may be more strongly influenced by membrane factors than tumor factors. It is well known that adhesion affects cell condition 37, 38, 39. In this study, SBC‐3A cells were affected not only by the extracellular matrix, but also by factors in the cell membrane, which in turn were regulated by cell density. These results lead to the not unreasonable conclusion that expression of u‐PA was induced when angiogenesis and metastasis were required.

Neuropeptides mature in intracellular environments such as the Golgi body and secretory vesicles. In some tumor and normal cells, cells released neuropeptides in precursor form 16, 17, 18, 40, 41. We previously demonstrated that progalanin released from SCLC was cleaved to galanin (1–20) by trypsin‐like protease, with plasmin as the major protease 25, 26, 27. Activated galanin induced expression of matrix metalloproteases‐2 and ‐9, following angiogenesis and metastasis 27. The present study showed that tranexamic acid suppressed hemoglobin content and the production of galanin (1–20) in tumor tissue. In addition, supplying galanin to the tumor resulted in the recovery of hemoglobin content. This result supports the previous report that shows galanin induced angiogenesis in tumor and granulation tissue 42, 43. Furthermore, our results supported the idea that the u‐PA‐plasminogen system regulates the mechanism behind progalanin activation, a reasonable conclusion given that u‐PA actively converts plasminogen to plasmin and plasmin affects angiogenesis and metastasis, including progalanin activation.

The present study showed that GALR2‐deficient SBC‐3A cells did not respond to galanin with an increase in hemoglobin content. This indicates that GALR2 plays an important role in increasing the hemoglobin content, implying that galanin affects angiogenesis via the GALR2 expressed in SCLC cells. There are some reports that GALR2 is expressed in tumors and plays a role in the acceleration or inhibition of tumor growth 44, 45, 46. The u‐PA/plasmin/galanin system is thus one mechanism for regulating tumor growth, and it appears to be an anomalous autocrine mechanism.

In summary, we demonstrated that cell membrane factors induce u‐PA expression. The expression of these factors increased with increasing cell density. In addition, u‐PA expression in tumors activated progalanin via plasminogen cleavage. Our results suggest that excessive tumor growth induces progalanin activation via the u‐PA/plasminogen system.

## Author contributions

HY and KU contributed to the experimental design. HY, RO, and YH performed the experiment. HY wrote the manuscript.

## Supporting information


**Fig. S1.** RT‐PCR.
**Fig. S2.** [Ca^2+^]i response to galanin in SBC‐3A and SBC‐3A‐Y cells.
