# Supplementary figures and images for: Expression of a urokinase‐type plasminogen activator during tumor growth leads to angiogenesis via galanin activation in tumor‐bearing mice
Source: FEBS Open Bio. 2017 Oct 9;7(11):1784–92. doi: 10.1002/2211-5463.12318 (PMC5666387; doi:10.1002/2211-5463.12318)

Fig. S1

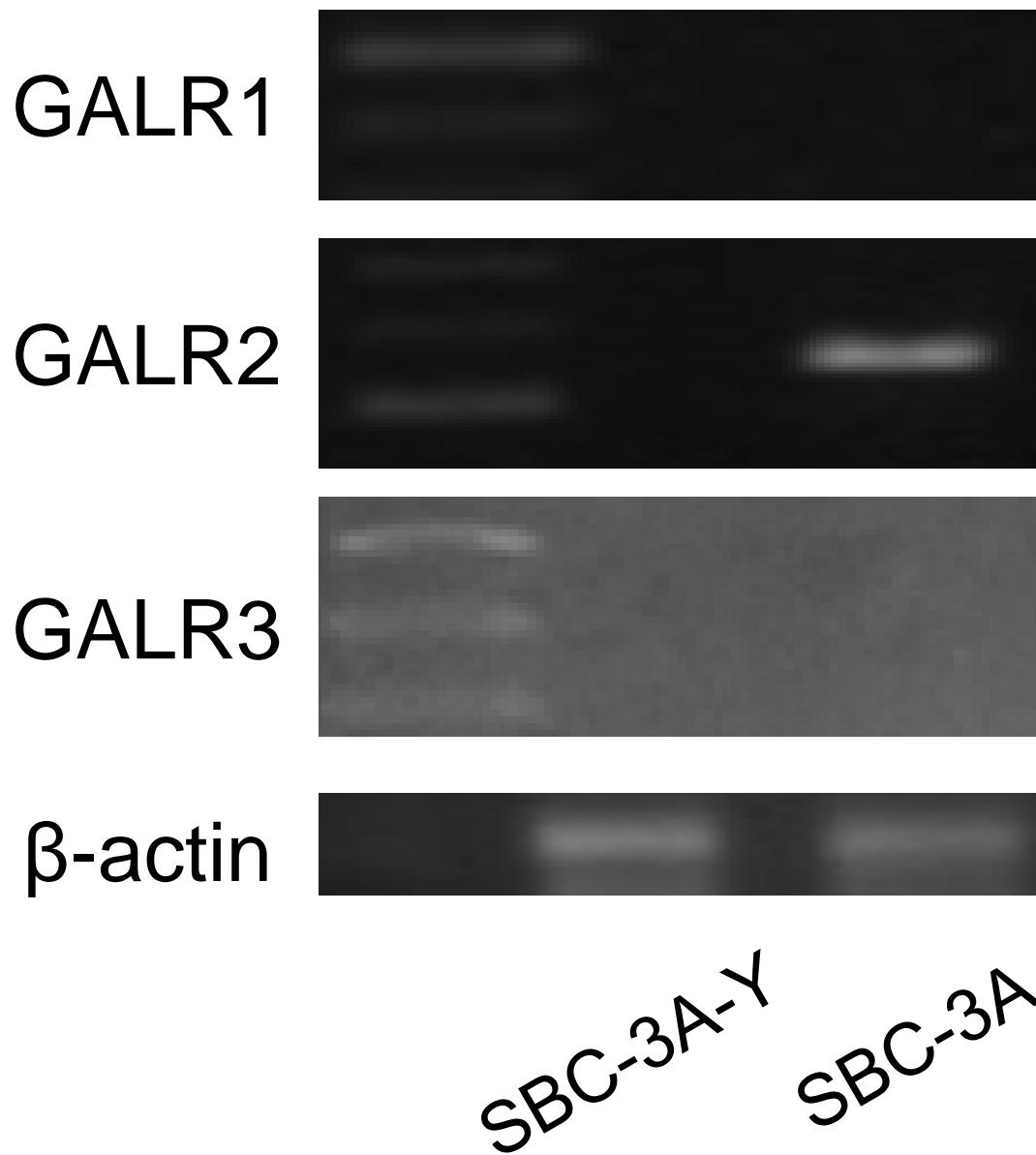

Fig. S2

**A** SBC-3A

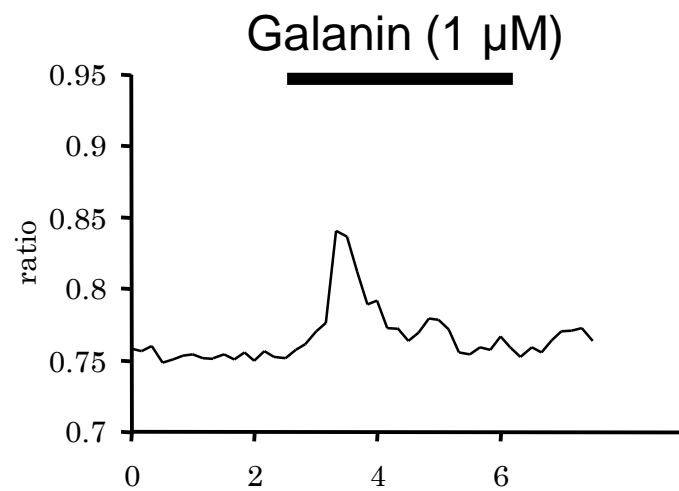

**B** SBC-3A-Y

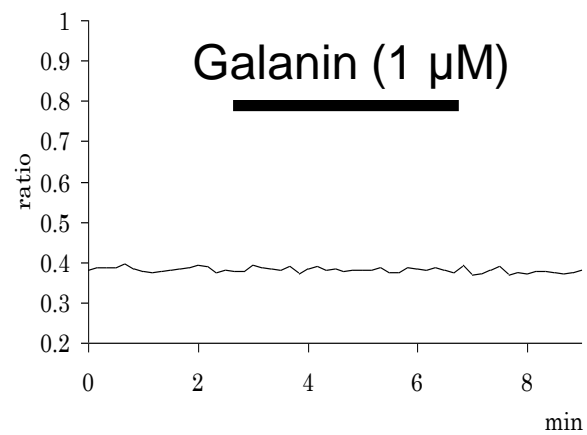

Supplement: Supplementary file 1 — Fig. S1. RT‐PCR. Fig. S2. [Ca2+]i response to galanin in SBC‐3A and SBC‐3A‐Y cells. [file FEB4-7-1784-s001.pdf]
